# Nuclear translocation of haeme oxygenase-1 is associated to prostate cancer

**DOI:** 10.1038/sj.bjc.6604081

**Published:** 2007-11-20

**Authors:** P Sacca, R Meiss, G Casas, O Mazza, J C Calvo, N Navone, E Vazquez

**Affiliations:** 1 Instituto de Biología y Medicina Experimental, CONICET, Vuelta de Obligado 2490, Buenos Aires 1428, Argentina; 2 Departamento de Patología, Instituto de Estudios Oncológicos, Academia Nacional de Medicina, Pacheco de Melo 3081, Buenos Aires 1425, Argentina; 3 Departamento de Patología, Hospital Alemán, Buenos Aires, Argentina; 4 Servicio Urología, Hospital de Clínicas, Universidad de Buenos Aires, Buenos Aires, Argentina; 5 Departamento de Química Biológica, Facultad de Ciencias Exactas y Naturales, Universidad de Buenos Aires, Ciudad Universitaria, CONICET, Pabellón II, 4to Piso, Buenos Aires 1428, Argentina; 6 Department of Genitourinary Medical Oncology, The University of Texas MD Anderson Cancer Center, Houston, TX, USA

**Keywords:** haeme oxygenase-1, prostate cancer, nuclear localisation, oxidative stress

## Abstract

The role of oxidative stress in prostate cancer has been increasingly recognised. Acute and chronic inflammations generate reactive oxygen species that result in damage to cellular structures. Haeme oxygenase-1 (HO-1) has cytoprotective effects against oxidative damage. We hypothesise that modulation of HO-1 expression may be involved in the process of prostate carcinogenesis and prostate cancer progression. We thus studied HO-1 expression and localisation in 85 samples of organ-confined primary prostate cancer obtained via radical prostatectomy (Gleason grades 4–9) and in 39 specimens of benign prostatic hyperplasia (BPH). We assessed HO-1 expression by immunohistochemical staining. No significant difference was observed in the cytoplasmic positive reactivity among tumours (84%), non-neoplastic surrounding parenchyma (89%), or BPH samples (87%) (*P*=0.53). Haeme oxygenase-1 immunostaining was detected in the nuclei of prostate cancer cells in 55 of 85 (65%) patients but less often in non-neoplastic surrounding parenchyma (30 of 85, 35%) or in BPH (9 of 39, 23%) (*P*<0.0001). Immunocytochemical and western blot analysis showed HO-1 only in the cytoplasmic compartment of PC3 and LNCaP prostate cancer cell lines. Treatment with hemin, a well-known specific inducer of HO-1, led to clear nuclear localisation of HO-1 in both cell lines and highly induced HO-1 expression in both cellular compartments. These findings have demonstrated, for the first time, that HO-1 expression and nuclear localisation can define a new subgroup of prostate cancer primary tumours and that the modulation of HO-1 expression and its nuclear translocation could represent new avenues for therapy.

Several factors are involved in the development of prostate cancer (PCa), such as age, genetic predisposition, environmental factors, diet, and exposure to infectious agents or androgens, which induce an imbalance in the redox state of the tissue (Malins *et al*, 2001; Fleshner *et al*, 2004; Calabrese and Maines, 2006; Klein *et al*, 2006). The role of oxidative stress in PCa has been increasingly recognised. The ultimate effect of these events is to produce tissue remodelling and proliferation (De Marzo *et al*, 2003). Acute and chronic inflammations generate reactive oxygen species that result in damage to cellular structures (Toyokuni *et al*, 1995).

Haeme oxygenase (HO) is the microsomal rate-limiting enzyme in haeme degradation (Tenhunen *et al*, 1968; Kikuchi *et al*, 2005). Haeme is the prosthetic moiety of various haeme proteins, including cytochrome *P*450. Haeme oxygenase-1 (HO-1) regulates cellular cytochrome *P*450 levels, which is related to steroidogenesis in prostate, and an inverse relationship between HO-1 activity and the level of cytochrome *P*450 has been established (Maines and Abrahamsson, 1996).

To date, three different isoforms of mammalian HO have been discovered, HO-1, HO-2 and HO-3, and these have distinct patterns of tissue-specific gene expression (Prawan *et al*, 2005). Haeme oxygenase-1 is an inducible and ubiquitous 32 KDa isoform highly expressed in the spleen and liver and normally found in very low levels in mammalian tissue (Maines and Gibbs, 2005). The upregulation of HO-1 has been recognised as an adaptive response to several stress stimuli (Willis *et al*, 1996; Maines, 2005; Morse and Choi, 2005). The regulation of its potent enzymatic activity depends primarily on the control of HO-1 expression at transcriptional level (Alam *et al*, 2004; Dulak *et al*, 2004). The role of HO-1 in tissue pathology is determined either by a delicate balance between the injurious and protective action of the end products generated during haeme catabolism (Dong *et al*, 2000) or by exerting a function distinct from haeme degradation (Vazquez *et al*, 2002) and playing a more proactive role in physiological and pathological processes (Willis, 1999; Maines, 2000). Hence, the induction of HO-1 is one of the most important events in cellular response to pro-oxidative and proinflammatory insults (Prawan *et al*, 2005).

Haeme oxygenase-1 has been detected in several cancer cell lines (Nishie *et al*, 1999; Chen *et al*, 2000; Liu *et al*, 2004; Busserolles *et al*, 2006) and tumours (Fang *et al*, 2003; Tanaka *et al*, 2003), but its role is still controversial. Recently, *in vivo* studies have proposed HO-1 upregulation as a useful marker in identifying patients with oral squamous cell carcinoma at low risk of metastasis (Tsuji *et al*, 1999) and as a novel BCR/ABL-dependent survival factor in chronic myeloid leukaemia (Mayerhofer *et al*, 2004). Moreover, Hill *et al* (2005) proposed that HO-1 exerts antitumour functions in rat and human breast cancer cells by antioxidant mechanisms. Targeted knockdown of HO-1 expression led to pronounced growth inhibition of pancreatic cancer cells and made tumour cells significantly more sensitive to radiotherapy and chemotherapy (Berberat *et al*, 2005). In human parotid pleomorphic adenomas, HO-1 may be implicated in these tumours (Lo *et al*, 2005).

Here, we assessed HO-1 expression and subcellular localisation in PCa specimens and tested if the expression/localisation profile correlates with PCa progression.

## MATERIALS AND METHODS

### Patients and tissue specimens

The use of tissue samples was approved by the Local Commission for Medical Ethics and Clinical Studies. All prostate tissues were obtained from the archival tissue bank of the Department of Pathology, Hospital Alemán, Buenos Aires, Argentina. Pathological specimens were taken from prostate radical prostatectomy specimens. Eighty-five PCa specimens were selected to represent the complete range of Gleason grades (Gleason, 1966). None of these patients received preoperative therapy. The age of the patients at the time of surgery and their preoperative PSA levels were recorded. Clinicopathological characteristics of the patients are shown in Table 1. In addition, 39 samples of benign prostatic hyperplasia (BPH) patients (59–76 years of age; mean 68 years), who underwent transurethral resection of the prostate, were included. All BPH specimens showed histological epithelial and/or stromal cell hyperplasia but no malignant cells.

### Antibodies

The following primary antibodies were used: rabbit polyclonal anti-haeme-oxygenase-1 (Stressgen Biotechnologies Corp., San Diego, CA, USA), mouse monoclonal anti-laminin A/C (Santa Cruz Biotechnology Inc., Santa Cruz, CA, USA) and mouse monoclonal anti-*β*-tubulin (Sigma, St Louis, MO, USA). Goat anti-rabbit and anti-mouse IgGs coupled to horseradish peroxidase were used as the secondary antibody (Santa Cruz Biotechnology Inc.).

### Immunohistochemical analysis

All tissues were processed and fixed using a routinely established protocol and stained as previously described (Caballero *et al*, 2004). Slides were counterstained with Mayer's haematoxylin and analysed by standard light microscopy. Sections incubated without primary antibody were used as negative controls.

Stained slides were examined and scored independently by two investigators (RM and GC). At least 20 randomly selected high-power fields with a minimum of 4000 cells were evaluated for expression both in the tumour and in the ‘normal’ tissue adjacent to the tumour (non-neoplastic surrounding parenchyma). The percentage of HO-1-positive cells was expressed as a ratio of positive cells to the total number of cells counted. We considered positive HO-1 expression when more than 25% cells exhibited positive cytoplasmic staining. Specimens with less than 25% of cells with cytoplasmic staining were considered negative. Nuclear HO-1 staining was considered positive when at least 5% of the cells demonstrated nuclear expression. Intraobserver error was calculated in a preliminary examination using the same material. It showed that at least 900 tumour cells should be assessed to have the results fall within 5% of the estimated real mean with a probability of 95%.

### Cell lines and reagents

LNCaP and PC3 cells were obtained from the American Type Culture Collection (Manassas, VA, USA) and were maintained at 37°C in a humidified incubator with a 5% CO_2_/95% air atmosphere in RPMI 1640 supplemented with 10% FCS. Culture reagents were obtained from Gibco BRL (Carlsbad, CA, USA). Hemin chloride (equine), protease inhibitor cocktail for mammalian tissue and phosphatase inhibitors (Na_3_VO_4_, NaF and Na_4_P_2_O_7_) were obtained from Sigma.

### Immunocytochemical analysis

LNCaP and PC3 cells were plated in Labtek chamber slides and incubated with and without hemin (20 *μ*M, 22 h). Then, slides were fixed in methanol (5 min, −20°C) and permeabilised with 0.2% Triton X-100 in PBS. After blocking with hydrogen peroxide and with 2% bovine serum albumin, cells were incubated with anti-HO-1 (1 : 5000), washed with PBS and incubated with the secondary antibody.

### Western blot analysis

For the isolation of nuclear and cytoplasmic fractions, LNCaP and PC3 cells were treated or not with hemin (20 *μ*M, 22 h) and lysed with low-salt buffer A (50 mM HEPES, 10 mM KCl, 1 mM EDTA, 1 mM EGTA, 1 mM DTT, cocktail protease and phosphatase inhibitors (1 mM Na_3_VO_4_, 20 mM NaF and 1 mM Na_4_P_2_O_7_), pH 7.9). After centrifugation, the cytoplasmic supernatant was separated and the nuclear pellet was gently resuspended in low-salt sucrose buffer (low-salt buffer A plus 1.0 M sucrose). After centrifugation, the nuclear pellet was vortexed for 20 min with high-salt extraction buffer (50 mM HEPES, 400 mM KCl, 1 mM EDTA, 1 mM EGTA, 1 mM DTT, and protease and phosphatase inhibitors, pH 7.9). Protein concentrations were determined in both fractions using a BCA procedure (Pierce Biochemical, Rockford, IL, USA). Western blot analysis was performed as previously described (Sacca *et al*, 2004).

### Statistical analysis

The results of the staining were analysed statistically with Graph Pad software. Statistical significance of differences between HO-1-positive staining and clinicopathologic features (Gleason grade) was assessed by Fisher's exact test. Contingency tables were analysed using the *χ*^2^ test and Fisher's exact probability test to compare positive HO-1 staining in tumour cells with those in the non-tumoral parenchyma and in BPH.

## RESULTS

### HO-1 cytoplasmic localisation is similar in clinical prostate cancer, non-neoplastic surrounding parenchyma, and benign prostatic hyperplasia

**Figure 1:**
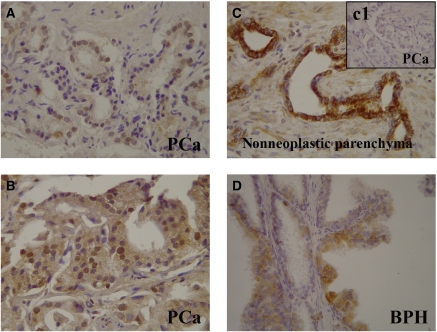
Immunohistochemical staining of HO-1 in human prostatic tissues. Representative findings of HO-1 immunoreactivity. (**A** and **B**) Nuclear/cytoplasmic staining in PCa samples. (**C**) Cytoplasmic staining of non-neoplastic parenchyma surrounding PCa with nuclear HO-1 exclusion. (**C1**) Negative nuclear/cytoplasmic staining in tumoral region of the same sample. (**D**) Papillar structure of BPH covered by HO-1-negative and HO-1-positive cells. Original magnification: × 40.

We found positive immunoreactivity for HO-1 in the cytoplasm of PCa cells, in epithelial cells of adjacent non-neoplastic areas and in epithelial cells of BPH (Figure 1A–D). Of the 85 cases with PCa analysed, we found positive cytoplasmic staining in 71 of 85 (84%) tissue sections with PCa and in 76 of 85 (89%) tissue section when evaluating areas of adjacent non-neoplastic specimens (Table 2). Also, 34 of 39 (87%) tissue samples from BPH were positive for HO-1 cytoplasmic staining (Table 2). Statistical analysis of these results found no significant difference in the cytoplasmic immunoreactivity between area with tumour, non-neoplastic surrounding parenchyma and in BPH samples (*χ*^2^, *P*=0.53). Positive HO-1 cytoplasmic immunoreactivity was seen in a small fraction of basal cells of the non-neoplastic surrounding tissue (7 of 85, 8%) and BPH (3 of 39, 8%). The degree of HO-1 expression in epithelial cells of PCa non-neoplastic surrounding parenchyma (89%) and BPH (87%) compare to basal cells (8 and 8%, respectively) was similar for both groups, reflecting a uniform HO-1 cytoplasmic localisation in both samples (Table 2).

### Haeme oxygenase-1 is expressed in the nuclei of prostate cancer cells in patient specimens

Haeme oxygenase-1 immunostaining was detected in the nuclei of PCa cells in 55 of 85 (65%) tissue specimens from PCa patients (Figure 1A and B; Table 2). The degree of HO-1 expression in the nuclei of PCa cells was significantly higher than in the nuclei of prostate epithelial cells in non-neoplastic surrounding parenchyma (30 of 85, 35%) or BPH (9 of 39, 23%) (*χ*^2^, *P*<0.0001) (Table 2). Moreover, the results also showed a quite positive correlation with Gleason score (Gleason 4–6, 53%; high Gleason 8–10, 76%); the higher the Gleason score, the more the number of samples with nuclear HO-1-positive staining (*P*=0.0983 considered quite significant) (Table 3). The rate of nuclear HO-1 expression in PCa non-tumour surrounding parenchyma was about 34% independent of the Gleason score of the samples (Table 3). Furthermore, relative risk factors for nuclear staining evaluated by Fisher's exact test were 1.8 tumour *vs* non-neoplastic surrounding parenchyma and 3.45 tumour *vs* BPH (Table 4). These results suggest that HO-1 nuclear expression is associated with malignant transformation.

### Hemin can induce nuclear translocation of HO-1 in PCa cells

**Figure 2:**
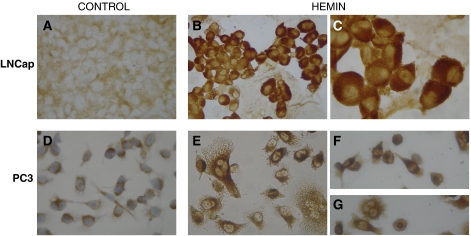
Immunohistochemical detection of HO-1 nuclear translocation induced by hemin. Cytoplasmic immunostaining in LNCaP (**A**) and PC3 (**D**) cells grown under control conditions. Positive nuclear staining in LNCaP (**B** and **C**) and PC3 (**E**–**G**) cells grown with hemin (20 *μ*M) during 22 h. Magnification: × 40 (**A**, **B**, **D**–**G**) and × 100 (**C**).

**Figure 3:**
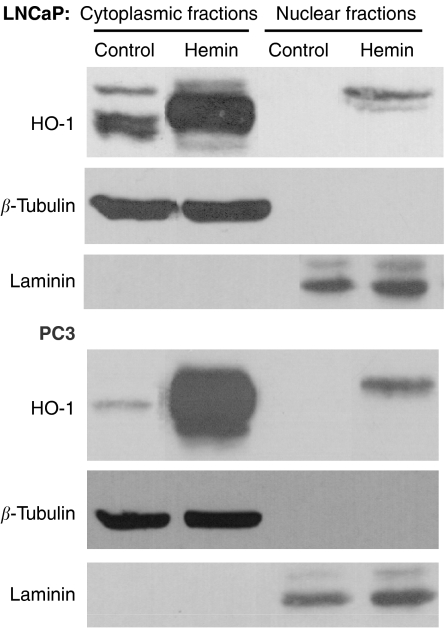
Hemin induces the nuclear translocation of HO-1 in LNCaP and PC3 cell lines. Western blot analysis of HO-1 (32 kDa) in nuclear and cytoplasmic fractions extracted from LNCaP and PC3 cells cultured with or without hemin (20 *μ*M) for 22 h. Equal loading of the samples was verified by the detection of *β*-tubulin (55 kDa) and laminin A/C (67 kDa).

Haeme oxygenase-1 was found in the cytoplasm of untreated PC3 or LNCaP cells, with clear nuclear exclusion (Figure 2A and D). Treatment with Hemin, a well-known specific inducer of HO-1, resulted in an increased-intensity HO-1 cytoplasmic staining and induction of nuclear localisation in both cell lines (Figure 2B, C, E–G). Western blot analysis of nuclear and cytoplasmic protein extracts from treated and untreated cells confirmed these findings (Figure 3). The purity of the cytoplasmic and nuclear fractions was verified in all samples by detection of *β*-tubulin and laminin A/C, respectively. Furthermore, basal cytoplasmic expression of HO-1 was lower in PC3 than in LNCaP. These results demonstrate that HO-1 expression and localisation could be modulated by hemin in androgen-insensitive and androgen-sensitive PCa cells.

## DISCUSSION

In this report, we have demonstrated that HO-1 nuclear localisation occurs in a subset of PCa. Haeme oxygenase-1 nuclear localisation is likely associated with carcinogenesis rather than with progression because it was only quite associated with Gleason score.

Although one previous report had shown increased HO-1 expression in localised prostate carcinoma and BPH, the small sample size of that study (six cases) precluded any conclusion on the relevance of these findings. The higher detection frequency of HO-1 expression and the more nuclear staining of HO-1 in our study compared with the results of Maines and Abrahamsson (1996) from a number of samples analysed covering all the ranges of PCa progression and several cases of BPH. Here, we report that whereas cytoplasmic HO-1 staining appears to correlate with moderate levels of HO-1 expression, high levels of the protein tend to correlate with a shift to nuclear translocation. Using immunocytochemistry techniques and western blot analysis, we confirmed HO-1 nuclear translocation either in androgen-dependent or androgen-independent PCa cells mediated by hemin induction (Figure 2, Figure 3).

It is believed that intracellular localisation of HO isoforms may be related to selective functions in different cell types (Parfenova *et al*, 2001). In particular, nuclear HO-1 localisation in astroglial cells was implicated in brain development and neurodegenerative diseases (Li Volti *et al*, 2004), in rat fetal lung cells exposed to hyperoxia as a chaperone or a nuclear messenger (Suttner *et al*, 1999) and in brown adipocyte as a transcription factor in adipogenesis (Giordano *et al*, 2000). Recently, HO-1 immunoreactive signal was detected in the nucleus of cultured cells after exposure to hypoxia and haeme, suggesting that this localisation may serve to upregulate genes that promote cytoprotection against oxidative stress (Lin *et al*, 2007). Although several studies have implicated HO-1 with cancer (Prawan *et al*, 2005), no report has associated this protein expression with its nuclear translocation.

Haeme plays an important role in activating the expression of different genes by regulation of various transcription factors. In response to haeme, these transcription factors bind to activation sequences of numerous genes encoding functions required for respiration and for controlling oxidative damage (Hon *et al*, 1999). As with other heat-shock proteins (Segui-Simarro *et al*, 2003), the transport of HO-1 could involve either interaction of the enzyme nuclear localisation signal with the nuclear pore complex or with other cytoplasmic components that would deliver the protein (Li Volti *et al*, 2004).

A number of transcriptional activators are regulated by redox modulation, including c-myb, Ets, early growth response-1, the glucocorticoid receptor, members of the activating transcription factor/cAMP-responsive element binding family, and HIF-1a (Guehmann *et al*, 1992; Huang and Adamson, 1993; Wasylyk and Wasylyk, 1993; Esposito *et al*, 1995; Huang *et al*, 1996).

Redox regulation is one of the key mechanisms for adapting to a variety of stresses, including oxidative stress (Goodman *et al*, 1997). Excess generation of ROS can cause DNA damage (Ohshima *et al*, 2003; Toyokuni, 2006), leading to changes in the genomic information in spite of the strong counteractions of repair enzymes and apoptotic pathways. The localisation of oxidative nucleic acid damage in certain specific sequences, which are especially vulnerable to oxidative stress, may differ depending on the cell type and cellular environment (Toyokuni, 2006). Such difference could explain the specific signalling pathway turns on/off in each type of cancer.

Where DNA damage is involved, HO-1 may emerge to counteract stress-induced apoptosis and represent a mutagenic/carcinogenic defence mechanism to protect cells expressing unrepairable DNA damage. It is possible that HO-1 may modulate proliferation by scavenging and/or preventing the formation of reactive oxygen metabolites. This is particularly relevant to active proliferating cells with low levels of antioxidant detoxifying enzymes. Haeme oxygenase-1 may therefore be one guardian of the genome, limiting mutations of DNA and promoting deletion of aberrant cells (Oates and West, 2006). An altered prooxidant/antioxidant balance in PCa patients, reflected by elevated lipid peroxidation and concomitant antioxidant depletion, was suggested to lead to an increase in oxidative damage playing an important role in prostate carcinogenesis (Yilmaz *et al*, 2004; Aydin *et al*, 2006).

Hence, HO-1 is found exclusively in the cytoplasm of some cells, and in others it is localised both in the cytoplasm and nucleus. The biological relevance of the compartmentalisation of HO-1 is not understood, but the complexity of staining patterns suggests that localisation is regulated.

Thus, if HO-1 seen in PCa cells is primarily functioning as an adaptive cellular defence system, its movement into the nucleus from the cytoplasm would have an impact on the ability of this protein to carry out other functions probably related to regulation of nuclear DNA repair activities, which could result in a different oncogenic phenotype.

Further studies that could correlate the HO-1 expression and nuclear translocation with the progression of the disease for each Gleason score could show a predictive value. In this way, it could be determined which patients with Gleason 5 or 6 could be considered for watchful and waiting and which ones for treatment, likewise in patients with Gleason 3+4 and 4+3 the convenience of radical prostatectomy or radiotherapy. However, HO-1 therapeutic implications in PCa are yet unclear.

## Figures and Tables

**Table 1:** Clinicopathological characteristics of prostate cancer patients

**Characteristic**	**Number of patients (%)**
Number of cases	85
	
*Age (years)*	
Range	44–92
Mean±s.d.	64±8.2
	
*Pathologic tumour category*	
pT1	15
pT2	40
pT3	30
	
*Gleason grade*	
4	3 (3.4)
5	17 (20.0)
6	18 (21.2)
7	26 (29.2)
8	15 (16.9)
9 and 10	6 (6.7)

**Table 2:** Immunohistochemical analysis of expression and localisation of positive HO-1 in patients with prostate cancer and benign prostatic hyperplasia

		**Tumour (*N*=85)**	**Non-neoplastic parenchyma (*N*=85)**	**Benign prostatic hyperplasia (*N*=39)**	
		**No. of cases (%)**	**No. of cases (%)**	**No. of cases (%)**	***P*-value**
Cytoplasm	Epithelial cells	71 (84)	76 (89)	34 (87)	0.53^a^
Cytoplasm	Basal cells	—	7 (8)	3 (8)	1.00^b^
Nucleus		55 (65)	30 (35)	9 (23)	<0.0001^a^

HO-1=haeme oxygenase-1.

a *χ*^2^ test for independence.

b Fisher's exact test.

**Table 3:** Relationship between positive nuclear HO-1 immunoreactivity and the Gleason score in prostate cancer human samples

	**Tumour**		**Non-neoplastic parenchyma**	
**Gleason score**	**No. of cases (%)**	***P*-value^a^**	**No. of cases (%)**	***P*-value^a^**
4–6 (*N*=38)	20 (53)		13 (34)	
7 (*N*=26)	19 (73)	0.123	10 (38)	0.79
8–10 (*N*=21)	16 (76)	0.0983^**^	7 (33)	1.00

HO-1=haeme oxygenase-1.

Fisher' s exact test.

a *P* *vs* Gleason 4–6.

^**^P-value considered not quite significant.

**Table 4:** Analysis of histological characteristics and positive HO-1 expression

**Histology**	**Relative risk**	**95% confidence interval^a^**	***P*-value^b^**
Tumour/BPH	3.45	1.88–6.35	<0.0001
Tumour/non-neoplastic parenchyma	1.83	1.32–2.55	0.0002
Non-neoplastic parenchyma/BPH	1.53	0.81–2.91	0.2138

BPH=benign prostatic hyperplasia; HO-1=haeme oxygenase-1.

a With Katz's approximation.

b Fisher's exact test.

## References

[bib1] Alam J, Igarashi K, Immenschuh S, Shibahara S, Tyrrell RM (2004) Regulation of heme oxygenase-1 gene transcription: recent advances and highlights from the International Conference (Uppsala, 2003) on Heme Oxygenase. Antioxid Redox Signal 6: 924–933 15345152 10.1089/ars.2004.6.924

[bib2] Aydin A, Arsova-Sarafinovska Z, Sayal A, Eken A, Erdem O, Erten K, Ozgok Y, Dimovski A (2006) Oxidative stress and antioxidant status in non-metastatic prostate cancer and benign prostatic hyperplasia. Clin Biochem 39: 176–179 16413012 10.1016/j.clinbiochem.2005.11.018

[bib3] Berberat PO, Dambrauskas Z, Gulbinas A, Giese T, Giese N, Kunzli B, Autschbach F, Meuer S, Buchler MW, Friess H (2005) Inhibition of heme oxygenase-1 increases responsiveness of pancreatic cancer cells to anticancer treatment. Clin Cancer Res 11: 3790–3798 15897578 10.1158/1078-0432.CCR-04-2159

[bib4] Busserolles J, Megias J, Terencio MC, Alcaraz MJ (2006) Heme oxygenase-1 inhibits apoptosis in Caco-2 cells via activation of Akt pathway. Int J Biochem Cell Biol 38: 1510–1517 16697692 10.1016/j.biocel.2006.03.013

[bib5] Caballero F, Meiss R, Gimenez A, Batlle A, Vazquez E (2004) Immunohistochemical analysis of heme oxygenase-1 in preneoplastic and neoplastic lesions during chemical hepatocarcinogenesis. Int J Exp Pathol 85: 213–222 15312126 10.1111/j.0959-9673.2004.00391.x PMC2517506

[bib6] Calabrese V, Maines MD (2006) Antiaging medicine: antioxidants and aging. Antioxid Redox Signal 8: 362–364 16677082 10.1089/ars.2006.8.362

[bib7] Chen X, Ding YW, Yang G, Bondoc F, Lee MJ, Yang CS (2000) Oxidative damage in an esophageal adenocarcinoma model with rats. Carcinogenesis 21: 257–263 10657966 10.1093/carcin/21.2.257

[bib8] De Marzo AM, Meeker AK, Zha S, Luo J, Nakayama M, Platz EA, Isaacs WB, Nelson WG (2003) Human prostate cancer precursors and pathobiology. Urology 62: 55–62 10.1016/j.urology.2003.09.053 14607218

[bib9] Dong Z, Lavrovsky Y, Venkatachalam MA, Roy AK (2000) Heme oxygenase-1 in tissue pathology: the Yin and Yang. Am J Pathol 156: 1485–1488 10793059 10.1016/S0002-9440(10)65019-5 PMC1876936

[bib10] Dulak J, Loboda A, Zagorska A, Jozkowicz A (2004) Complex role of heme oxygenase-1 in angiogenesis. Antioxid Redox Signal 6: 858–866 15345146 10.1089/ars.2004.6.858

[bib11] Esposito F, Cuccovillo F, Morra F, Russo T, Cimino F (1995) DNA binding activity of the glucocorticoid receptor is sensitive to redox changes in intact cells. Biochim Biophys Acta 1260: 308–314 7873605 10.1016/0167-4781(94)00209-l

[bib12] Fang J, Sawa T, Akaike T, Akuta T, Sahoo SK, Khaled G, Hamada A, Maeda H (2003) *In vivo* antitumor activity of pegylated zinc protoporphyrin: targeted inhibition of heme oxygenase in solid tumor. Cancer Res 63: 3567–3574 12839943

[bib13] Fleshner N, Bagnell PS, Klotz L, Venkateswaran V (2004) Dietary fat and prostate cancer. J Urol 171: S19–S24 14713748 10.1097/01.ju.0000107838.33623.19

[bib14] Giordano A, Nisoli E, Tonello C, Cancello R, Carruba MO, Cinti S (2000) Expression and distribution of heme oxygenase-1 and -2 in rat brown adipose tissue: the modulatory role of the noradrenergic system. FEBS Lett 487: 171–175 11150503 10.1016/s0014-5793(00)02217-1

[bib15] Gleason DF (1966) Classification of prostatic carcinomas. Cancer Chemother Rep 50: 125–128 5948714

[bib16] Goodman AI, Choudhury M, da Silva JL, Schwartzman ML, Abraham NG (1997) Overexpression of the heme oxygenase gene in renal cell carcinoma. Proc Soc Exp Biol Med 214: 54–61 9012361 10.3181/00379727-214-44069

[bib17] Guehmann S, Vorbrueggen G, Kalkbrenner F, Moelling K (1992) Reduction of a conserved Cys is essential for Myb DNA-binding. Nucleic Acids Res 20: 2279–2286 1594446 10.1093/nar/20.9.2279 PMC312342

[bib18] Hill M, Pereira V, Chauveau C, Zagani R, Remy S, Tesson L, Mazal D, Ubillos L, Brion R, Asghar K, Mashreghi MF, Kotsch K, Moffett J, Doebis C, Seifert M, Boczkowski J, Osinaga E, Anegon I (2005) Heme oxygenase-1 inhibits rat and human breast cancer cell proliferation: mutual cross inhibition with indoleamine 2,3-dioxygenase. FASEB J 19: 1957–1968 16319139 10.1096/fj.05-3875com

[bib19] Hon T, Hach A, Tamalis D, Zhu Y, Zhang L (1999) The yeast heme-responsive transcriptional activator Hap1 is a preexisting dimer in the absence of heme. J Biol Chem 274: 22770–22774 10428861 10.1074/jbc.274.32.22770

[bib20] Huang LE, Arany Z, Livingston DM, Bunn HF (1996) Activation of hypoxia-inducible transcription factor depends primarily upon redox-sensitive stabilization of its alpha subunit. J Biol Chem 271: 32253–32259 8943284 10.1074/jbc.271.50.32253

[bib21] Huang RP, Adamson ED (1993) Characterization of the DNA-binding properties of the early growth response-1 (Egr-1) transcription factor: evidence for modulation by a redox mechanism. DNA Cell Biol 12: 265–273 8466649 10.1089/dna.1993.12.265

[bib22] Kikuchi G, Yoshida T, Noguchi M (2005) Heme oxygenase and heme degradation. Biochem Biophys Res Commun 338: 558–567 16115609 10.1016/j.bbrc.2005.08.020

[bib23] Klein EA, Casey G, Silverman R (2006) Genetic susceptibility and oxidative stress in prostate cancer: integrated model with implications for prevention. Urology 68: 1145–1151 17169635 10.1016/j.urology.2006.08.1074

[bib24] Li Volti G, Ientile R, Abraham NG, Vanella A, Cannavo G, Mazza F, Curro M, Raciti G, Avola R, Campisi A (2004) Immunocytochemical localization and expression of heme oxygenase-1 in primary astroglial cell cultures during differentiation: effect of glutamate. Biochem Biophys Res Commun 315: 517–524 14766239 10.1016/j.bbrc.2004.01.090

[bib25] Lin Q, Weis S, Yang G, Weng YH, Helston R, Rish K, Smith A, Bordner J, Polte T, Gaunitz F, Dennery PA (2007) Heme oxygenase-1 protein localizes to the nucleus and activates transcription factors important in oxidative stress. J Biol Chem 282: 20621–20633 17430897 10.1074/jbc.M607954200

[bib26] Liu ZM, Chen GG, Ng EK, Leung WK, Sung JJ, Chung SC (2004) Upregulation of heme oxygenase-1 and p21 confers resistance to apoptosis in human gastric cancer cells. Oncogene 23: 503–513 14647439 10.1038/sj.onc.1207173

[bib27] Lo S, Di PS, Yusuf H, McCombe AW (2005) Constitutive (HO-2) and inducible (HO-1) haem oxygenase in pleomorphic adenomas of the human parotid: an immunocytochemical study. J Laryngol Otol 119: 179–183 15845187 10.1258/0022215053561567

[bib28] Maines MD (2000) The heme oxygenase system and its functions in the brain. Cell Mol Biol (Noisy-le-grand) 46: 573–585 10872744

[bib29] Maines MD (2005) The heme oxygenase system: update 2005. Antioxid Redox Signal 7: 1761–1766 16356137 10.1089/ars.2005.7.1761

[bib30] Maines MD, Abrahamsson PA (1996) Expression of heme oxygenase-1 (HSP32) in human prostate: normal, hyperplastic, and tumor tissue distribution. Urology 47: 727–733 8650873 10.1016/s0090-4295(96)00010-6

[bib31] Maines MD, Gibbs PE (2005) 30 some years of heme oxygenase: from a ‘molecular wrecking ball’ to a ‘mesmerizing’ trigger of cellular events. Biochem Biophys Res Commun 338: 568–577 16183036 10.1016/j.bbrc.2005.08.121

[bib32] Malins DC, Johnson PM, Wheeler TM, Barker EA, Polissar NL, Vinson MA (2001) Age-related radical-induced DNA damage is linked to prostate cancer. Cancer Res 61: 6025–6028 11507046

[bib33] Mayerhofer M, Florian S, Krauth MT, Aichberger KJ, Bilban M, Marculescu R, Printz D, Fritsch G, Wagner O, Selzer E, Sperr WR, Valent P, Sillaber C (2004) Identification of heme oxygenase-1 as a novel BCR/ABL-dependent survival factor in chronic myeloid leukemia. Cancer Res 64: 3148–3154 15126353 10.1158/0008-5472.can-03-1200

[bib34] Morse D, Choi AM (2005) Heme oxygenase-1: from bench to bedside. Am J Respir Crit Care Med 172: 660–670 15901614 10.1164/rccm.200404-465SO

[bib35] Nishie A, Ono M, Shono T, Fukushi J, Otsubo M, Onoue H, Ito Y, Inamura T, Ikezaki K, Fukui M, Iwaki T, Kuwano M (1999) Macrophage infiltration and heme oxygenase-1 expression correlate with angiogenesis in human gliomas. Clin Cancer Res 5: 1107–1113 10353745

[bib36] Oates PS, West AR (2006) Heme in intestinal epithelial cell turnover, differentiation, detoxification, inflammation, carcinogenesis, absorption and motility. World J Gastroenterol 12: 4281–4295 16865768 10.3748/wjg.v12.i27.4281 PMC4087737

[bib37] Ohshima H, Tatemichi M, Sawa T (2003) Chemical basis of inflammation-induced carcinogenesis. Arch Biochem Biophys 417: 3–11 12921773 10.1016/s0003-9861(03)00283-2

[bib38] Parfenova H, Neff III RA, Alonso JS, Shlopov BV, Jamal CN, Sarkisova SA, Leffler CW (2001) Cerebral vascular endothelial heme oxygenase: expression, localization, and activation by glutamate. Am J Physiol Cell Physiol 281: C1954–C1963 11698254 10.1152/ajpcell.2001.281.6.C1954

[bib39] Prawan A, Kundu JK, Surh YJ (2005) Molecular basis of heme oxygenase-1 induction: implications for chemoprevention and chemoprotection. Antioxid Redox Signal 7: 1688–1703 16356130 10.1089/ars.2005.7.1688

[bib40] Sacca P, Caballero F, Batlle A, Vazquez E (2004) Cell cycle arrest and modulation of HO-1 expression induced by acetyl salicylic acid in hepatocarcinogenesis. Int J Biochem Cell Biol 36: 1945–1953 15203109 10.1016/j.biocel.2004.01.029

[bib41] Segui-Simarro JM, Testillano PS, Risueno MC (2003) Hsp70 and Hsp90 change their expression and subcellular localization after microspore embryogenesis induction in *Brassica napus* L. J Struct Biol 142: 379–391 12781665 10.1016/s1047-8477(03)00067-4

[bib42] Suttner DM, Sridhar K, Lee CS, Tomura T, Hansen TN, Dennery PA (1999) Protective effects of transient HO-1 overexpression on susceptibility to oxygen toxicity in lung cells. Am J Physiol 276: L443–L451 10070108 10.1152/ajplung.1999.276.3.L443

[bib43] Tanaka S, Akaike T, Fang J, Beppu T, Ogawa M, Tamura F, Miyamoto Y, Maeda H (2003) Antiapoptotic effect of haem oxygenase-1 induced by nitric oxide in experimental solid tumour. Br J Cancer 88: 902–909 12644828 10.1038/sj.bjc.6600830 PMC2377071

[bib44] Tenhunen R, Marver HS, Schmid R (1968) The enzymatic conversion of heme to bilirubin by microsomal heme oxygenase. Proc Natl Acad Sci USA 61: 748–755 4386763 10.1073/pnas.61.2.748 PMC225223

[bib45] Toyokuni S (2006) Novel aspects of oxidative stress-associated carcinogenesis. Antioxid Redox Signal 8: 1373–1377 16910784 10.1089/ars.2006.8.1373

[bib46] Toyokuni S, Okamoto K, Yodoi J, Hiai H (1995) Persistent oxidative stress in cancer. FEBS Lett 358: 1–3 7821417 10.1016/0014-5793(94)01368-b

[bib47] Tsuji MH, Yanagawa T, Iwasa S, Tabuchi K, Onizawa K, Bannai S, Toyooka H, Yoshida H (1999) Heme oxygenase-1 expression in oral squamous cell carcinoma as involved in lymph node metastasis. Cancer Lett 138: 53–59 10378773 10.1016/s0304-3835(98)00372-3

[bib48] Vazquez E, Gerez E, Caballero F, Olivieri L, Falcoff N, Tomaro ML, Batlle A (2002) On the promoting action of tamoxifen in the *p*-dimethylaminoazobenzene induced hepatocarcinogenesis CF1 mice model and the cytoprotective role of heme oxygenase. In Heme Oxygenase in Biology and Medicine, Abraham NG, Alan J, Nath K (eds), pp 469–479. New York, Boston, Dordrecht, London, Moscow: Kluwer Academic/Plenum

[bib49] Wasylyk C, Wasylyk B (1993) Oncogenic conversion of Ets affects redox regulation *in-vivo* and *in-vitro*. Nucleic Acids Res 21: 523–529 8441665 10.1093/nar/21.3.523 PMC309148

[bib50] Willis D (1999) Overview of HO-1 in inflammatory pathologies. In Inducible Enzymes in the Inflammatory Response, Willoughby DA, Tomlinson A (eds), pp 55–92. Basel: Birkauser Verlag

[bib51] Willis D, Moore AR, Frederick R, Willoughby DA (1996) Heme oxygenase: a novel target for the modulation of the inflammatory response. Nat Med 2: 87–90 8564848 10.1038/nm0196-87

[bib52] Yilmaz MI, Saglam K, Sonmez A, Gok DE, Basal S, Kilic S, Akay C, Kocar IH (2004) Antioxidant system activation in prostate cancer. Biol Trace Elem Res 98: 13–19 15051896 10.1385/BTER:98:1:13

